# Signalling Pathways Implicated in Early Mammary Gland Morphogenesis and Breast Cancer

**DOI:** 10.1371/journal.pgen.0020112

**Published:** 2006-08-25

**Authors:** Beatrice Howard, Alan Ashworth

**Affiliations:** University College London, United Kingdom

## Abstract

Specification of mammary epithelial cell fate occurs during embryogenesis as cells aggregate to form the mammary anlage. Within the embryonic mammary bud, a population of epithelial cells exists that will subsequently proliferate to form a ductal tree filling the stromal compartment, and which can produce milk upon terminal differentiation after birth. Subsequently, these structures can be remodelled and returned to a basal state after weaning before regenerating in future pregnancies. The plasticity of the mammary epithelial cell, and its responsiveness to hormone receptors, facilitates this amazing biological feat, but aberrant signalling may also result in unintended consequences in the form of frequent malignancies. Reflecting this intimate connection, a considerable number of signalling pathways have been implicated in both mammary gland morphogenesis and carcinogenesis.

## Introduction

Like many other organs, mammary glands are formed by an exchange of signals between epithelia and mesenchyme [1–3]. However, the processes by which undifferentiated tissue is directed to form the mammary epithelial bud during embryogenesis have yet to be fully elucidated. It is unknown precisely how the mammary phenotype is conferred, but it appears that mesenchymal signals cause local migration of epidermal cells to form the mammary anlage rather than via localised proliferation [4–6]. As in the development of all epithelial appendages, pluripotent epidermal cells are directed along specific lineages so that a specialised structure, in this case, the mammary gland, forms [7,8]. The fundamental processes required for the inductive events of mammary bud development (epithelial migration, changes in cell adhesion, growth, death, and differentiation) are also those that are perturbed in breast cancers [9,10]. Characterizing inductive signalling pathways involved in mammary specification and patterning and the regulatory molecules that modulate this process will define potential targets of breast carcinogenesis. Here, we highlight the earliest signals mediating mammary specification and attempt to consolidate the current understanding of how mesenchymal factors act to bring about the initial stages of mammary anlage development. The relationship of the signalling pathways involved with those dysregulated in cancer will be discussed. Events during the later stages of mammary morphogenesis have recently been extensively reviewed [11–14].

## Mouse Mammary Anlage Development

**Figure 1:**
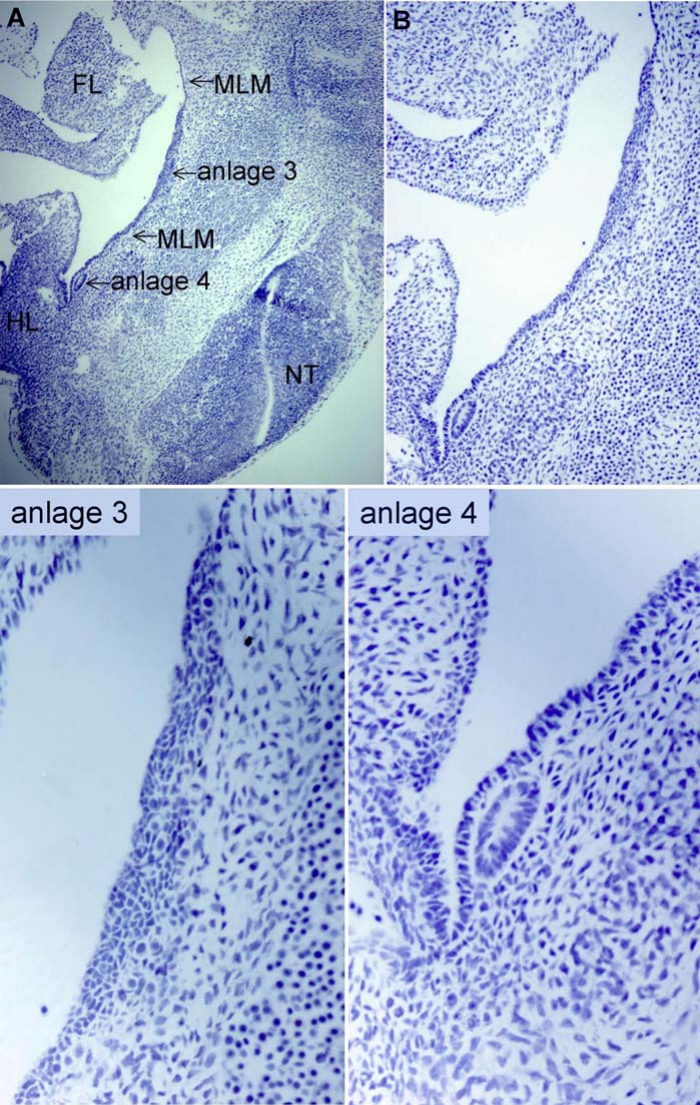
Mammary Anlagen (A) Mammary anlagen 3 and 4 are visible at E10.75/E11.0 (38–40 somites) in histological sagittal sections of mouse embryos stained with hematoxylin. FL, forelimb bud; HL, hindlimb bud; MLM, mammary line mesenchyme; NT, neural tube (B) Higher magnification of mammary region from (A). Anlagen 3 and 4 at higher magnification.

**Figure 2:**
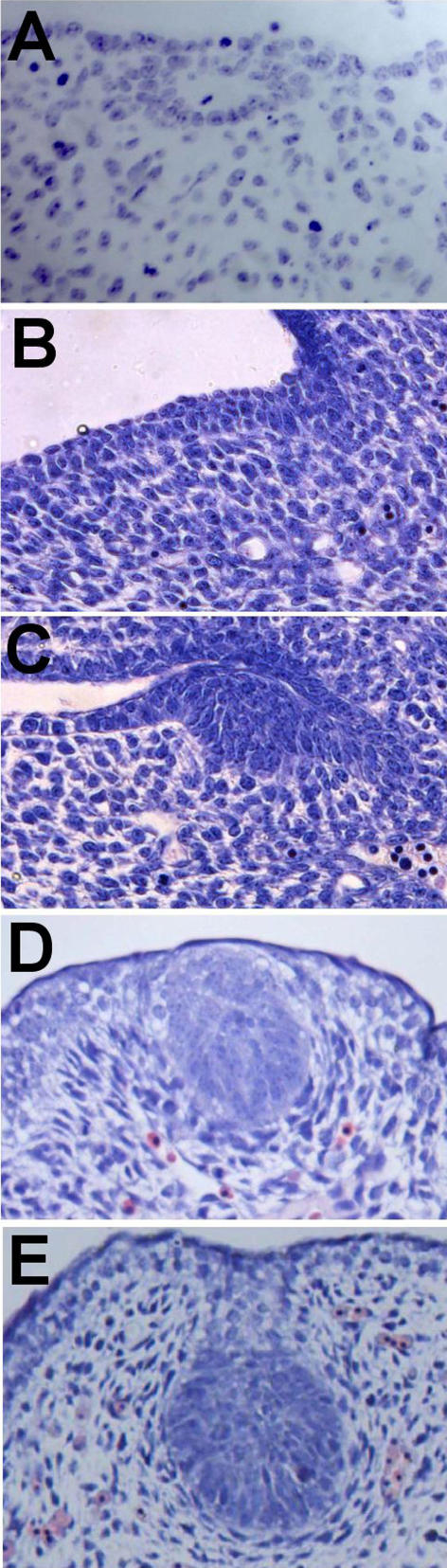
Histological Sections of Mammary Anlage 4 Stained with Hematoxylin (A) Late E10/E11 as cells initially aggregate into elliptical shape. (B) E11.5 epidermal thickening. (C) E12.0 at hillock stage. (D) E13.0 at spherical bud stage. (E) E14.5 at light bulb stage.

Mouse mammary epithelial buds normally form at distinct positions along the body axis and are easily visualised by staining for expression of mammary bud markers such as *Lef1,* a downstream Wnt signalling component, or by light microscopic analysis at E12.5. Histological analysis of sagittal sections reveals the first appearance of the mouse mammary anlage in late E10/early E11 embryos [6] (Figure 1A). At this time, distinct elliptical aggregates of epithelial cells are visible at the future site of mammary buds 3 and 4 as determined by their positions relative to the somites (Figure 1B). However, scanning electron microscopy reveals no overt indication of mammary anlage formation [14] at this stage. By E11.5 (~48 somite stage), anlage 3 protrudes and is observed by scanning electron microscopy as a slight elevation above the epidermal surface [15]. This is the first mammary anlagen pair detectable at the whole embryo level, with anlagen 4 becoming apparent shortly after anlagen 3. Pseudostratified epithelium is visible when cross sections through the future site of bud 3 are analysed at similar stages [6,16]. Since little localised proliferation occurs in the presumptive mammary region, it is assumed that epithelial cells locally migrate or aggregate to form the anlage, as is observed in rabbit embryos where a raised ridge of epithelial cells is apparent which have properties of motile cells such as lamellopodia [4–6,17]. After the elliptical shaped anlage appears, the cells become organised into a bud (Figure 2A–2E). The other anlagen (1,2,5) become morphologically distinct slightly later, and by E12 (52 somites) all five pairs of mammary buds are distinct/visible.

## Mammary Stem Cells

Within the anlage exists a population of epithelial cells, stem cells, with a capacity to self-renew and to generate daughter cells that can differentiate down distinct cell lineages such that all cell types of the mature mammary gland can form. Mammary epithelial buds can be isolated and transplanted into fat pads, which have been “cleared” of all epithelial ductal elements [18]. This technique is used experimentally to examine mammary ductal outgrowth in mutant mouse models that exhibit embryonic lethality and which would not otherwise be amenable to mammary gland phenotype analysis [19,20]. Mammary buds explanted at E12.5 routinely produce a ductal tree with all normal features of the mammary gland, except for the absence of a nipple. These experiments support the existence of a defined mammary stem cell population coincident with the appearance of the mammary bud at E12.5.

In the adult, a mammary stem cell population has recently been purified, but the relationship of this postnatal cell population with the “embryonic” stem cells has yet to be established [21,22]. Along with the potential for self-renewal, mammary epithelial stem cells are thought to harbour inherent tumourigenic potential as subsequent mammary development proceeds and continues postnatally. As DNA damage and mutations may accumulate throughout postnatal development, those progenitor cells adversely affected by genetic alterations can persist over the lifespan of an individual. This effectively creates a population of cells that harbour potentially deleterious genetic changes, instabilities, and modifications—the putative breast cancer stem cells [23,24]. How mammary progenitor cells attain the features that allow them to consistently confer mammary identity to their progeny and how the mammary phenotype is maintained remain to be addressed, but these processes begin during embryonic mammary morphogenesis.

## Mammary Mesenchyme Can Confer Mammary Epithelial Cell Fate

The exact time point at which the mammary epithelia attain regenerative capacity has not yet been determined, but it is likely to coincide with specification of the mammary anlage. Epithelium isolated from mammary glands at all subsequent stages retains the ability to generate ductal outgrowth [25,26]. An ongoing requirement for the maintenance of mammary cell fate beyond the formation of the anlage and bud stages is indicated from investigation of several mouse models that form mammary buds that do not progress beyond the bud stage [27,28]. In addition, reversion from the mammary phenotype can occur until E15.5, as shown in studies of inducible *PthrP* mouse models [29]. By E13, the embryonic mammary epithelium has been determined, and by E17, this becomes committed to the mammary fate as demonstrated by tissue recombination studies [30,31]. It should also be noted that, in addition to the promotion of mammary placode fate, there are likely to be factors that inhibit mammary placode fate. The fate of neighbouring cells is likely to be determined by the existence of an adjacent mammary anlage.

Tissue recombination experiments have demonstrated the inductive capability of the mammary mesenchyme [1,32,33]. In these experiments, epithelium from various nonmammary locations (dorsal, ventral) were induced to form mammary tissue when recombined with presumptive mammary mesenchyme (from rabbit embryos which form a raised epidermal ridge, the mammary line, prior to the formation of the anlage [33]) and mammary mesenchyme (from mouse, rat, or rabbit embryonic mesenchyme adjacent to the mammary epithelial bud) [14,32,34]. These results indicate that the mesenchymal cells along the mammary line have signalling/inductive properties, as does the mammary mesenchyme associated with the mammary buds. The secondary mammary mesenchyme (the future fat pad) does not have this inductive ability. This suggests that a window exists in which inductive properties exist within the mammary mesenchyme. Hyperplastic growth occurs when embryonic mammary mesenchyme is recombined with postnatal mammary epithelia [31,35]. This demonstrates the potentially detrimental influence of embryonic mammary regulators on postnatal mammary epithelial populations.

## Major Signalling Regulators of Mammary Anlage Formation Also Have Roles in Breast Cancer

**Figure 3:**
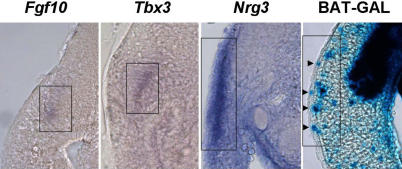
Expression of the Major Regulators of Mammary Anlage Formation 60-μm vibratome cross sections through the site where anlage 3 will subsequently form. *Fgf10* and *Tbx3* expression is observed in the dermamyotome along the mammary line of E10.5/32 somite stage B6 embryos. These expression patterns are maintained until the anlage becomes morphologically distinct. *Nrg3* is broadly expressed in the lateral plate mesoderm adjacent to the somite of E10.75 37 somite stage B6 embryos underlying the future site of anlage 3. Wnt expression (visualized using the BAT-GAL line of Wnt reporter mice [124]) is observed in the somite and by the future mammary anlage 3 site of E10.75 37 somite stage embryo. Both epithelial (arrowheads) and mesenchymal Wnt expression is observed along the mammary line. The expression along the presumptive mammary line is boxed.

Several mesenchymal signals have recently been identified that regulate the initial stages of mammary gland development and bring about local migration and changes in cell adhesion of epithelial cells (Figure 3). For example, Fgf10 signalling through Fgfr2b is required for mammary bud initiation with the exception of mammary bud 4, which can form in the absence of ligand or receptor [15]. MMTV-mediated insertional mutagenesis identified Fgfs as frequent mutational events in retrovirally induced mouse mammary tumours; activation of *Fgf3, Fgf4,* and *Fgf8* cooperates with Wnt signals in mouse mammary mammary tumourigenesis [36,37]. Moreover, *Fgf10* can also act as an oncogene in mice [38]. Increased levels of *FGF10* are observed in ~10% of human breast cancers [38], and amplification and overexpression of several FGFRs, including *FGFR1, FGFR2,* and *FGFR4,* have been observed in breast cancers [39–44]. FGFRs have well-characterised roles in angiogenesis and cell migration [45,46], and FGFR signalling promotes proliferation of breast cancer cells [47]. In addition to promoting proliferation, Fgfr1 signalling contributes to loss of cell polarity and the promotion of invasive properties such as *Mmp3* induction in a three-dimensional in vitro model of mouse mammary epithelial HC11 cells [48]. In transgenic mice, sustained FGFR1 activation induces alveolar hyperplasia and invasive mammary lesions [49]. Moreover, blocking FGFR signalling with a selective inhibitor of FGFR tyrosine kinase activity inhibits breast cancer cell proliferation through downregulation of several members of the CyclinD family [47]. As high levels of CyclinD1 are thought to contribute directly to tumourigenicity, inhibiting FGFR signalling is likely to be a useful therapeutic approach for some breast cancers.

Tbx3 is a transcriptional repressor that belongs to the Tbx2/3/4/5 subfamily of T-box transcriptional regulators [50]. *TBX3* is mutated in Ulnar Mammary Syndrome, a human disorder that disrupts apocrine gland and limb development [51]. The *Tbx3* mouse knockout model demonstrates a requirement for *Tbx3* for mammary bud initiation, with the minor caveat that occasionally one single mammary bud may form in these mice [52]. Signalling through Fgfr1 has been implicated in the induction of *Tbx3* expression [53]. Another intriguing role for Tbx3 has been demonstrated using *Tbx3* retrovirally delivered to chick embryos [54]. These experiments suggest that along with *dHand* and *Gli3,* Tbx3 can modulate the position of the limb buds along the anterior–posterior axis [54]. Although speculative, it is an attractive idea that a similar mechanism might operate in genesis of the mammary gland whereby Tbx3 and other factors determine the future site of the mammary buds along the body axis.

*TBX3* is overexpressed in some breast cancer cell lines [55], and high levels of expression of a truncated form of TBX3 are found in the plasma of early stage breast cancer patients [56]. Like *Tbx3, Tbx2* is initially expressed in the mesenchyme along the presumptive mammary line, prior to the formation of the anlage. *Tbx3* (but not *Tbx2*) is expressed in the epithelial compartment of the nascent anlagen [57]. Along with Tbx2, Tbx3 can repress senescence genes by inactivating the p53 response pathway [55]. The p19(ARF)-Mdm2-p53 pathway regulates the cell cycle and protects cells against oncogenic transformation, and Tbx3 strongly represses expression of both mouse p19(ARF) and human p14(ARF) [58]. Although *Tbx2*-null mice display no defects in the initiation of mammary development, placode maintenance defects are more severe in double heterozygotes for *Tbx2* and *Tbx3* than in *Tbx3* heterozygote mice [57]. This study also showed that during early mammary bud development the interaction of Tbx2 and Tbx3 is mediated via a p19Arf/p53-independent pathway.

Wnt signals are critical for mammary gland induction, and transgenic mice expressing the Wnt antagonist *Dkk1,* in developing epithelia, produce no mammary buds [59]. *Lef1* is required for mammary anlagen 2 and 3 formation [60]. The other anlagen, (1, 4, and 5) form in *Lef1*-null mice and then fail to progress beyond the E13.5 bud stage. Aggregates of Wnt-expressing epithelial and mesenchymal cells are apparent in the presumptive mammary region in E10.5 embryos [17,61]. The mammary placodes appear to be formed from aggregation of epithelial cells expressing at least one Wnt, including *Wnt10b,* which appears to connect the forming anlagen 2, 3, 4, and 5 along the milk line [16,17,62]. It seems plausible that these Wnt-expressing/Lef1-responsive cells are targets of the mesenchymal signals generated by *Tbx3* and *Fgf10* along the presumptive mammary line or that these signals induce Wnt expression in these epithelial cells along the sites where the mammary anlage form. However, the identity of the *Wnt* or *Wnts* involved in mammary inductive events is unknown, as is whether *Wnt* expression is required in either the epithelial or the mesenchymal cells or in both [17].

The Wnts that are likely to be involved in mammary specification and early morphogenesis, such as *Wnt3a, Wnt6,* and *Wnt10b,* are also genetically altered in MMTV-induced mammary tumours [17,63]. *WNT3A, WNT4, WNT6, WNT8B, WNT9A,* and *WNT10B* all are overexpressed in many breast cancer cell lines [64]. These WNTs signal through the canonical WNT/β-catenin signalling pathway. β-catenin and CyclinD1 overexpression is observed in some breast cancer cell lines and in a large percentage of breast cancers, but not in human mammary epithelial cells, which suggests that canonical WNT/β-catenin signalling is activated during carcinogenesis [65]. *WNT1, WNT4,* and the Wnt pathway components *AXIN2* and *LEF1* are upregulated in breast cancers [66]. The Frizzled 1 and 2 receptors (FZD1 and FZD2) are also overexpressed in breast cancer [67], and high β-catenin activity is significantly correlated with poor prognosis in breast cancer patients [65]. Increasing WNT1 signalling in human breast epithelial cells triggers the DNA damage response and promotes tumourigenic conversion through a Notch-dependent process [66]. Although mutations in upstream WNT signalling components have not been observed in breast cancers, inactivating mutations of *APC* are observed in some human breast tumours, and these likely increase the stability of β-catenin [68]. Although no such human mutations have been reported, mouse models that express stabilised β-catenin (by mutating the N-terminal domain) in either luminal or myoepithelial mammary cells form mammary carcinomas [69,70].

WNT antagonists may act as tumour suppressors and cause constitutive activation of WNT signalling when mutated; reduced expression of the secreted WNT inhibitors *SFRP1* and *WIF1* have been observed in breast cancers [71–73]. A recent study of 24 primary breast cancers showed that 67% were aberrantly methylated in the WIF1 promoter; this correlated with decreased expression in tumour samples when compared with normal tissue [73]. Downregulation of *SFRP1* expression is also observed in a significant proportion of invasive breast cancers and is frequently due to aberrant promoter hypermethylation [74,75]. *SFRP1* inactivation in breast cancer is associated with poor prognosis [75,76].

Both the *MMTV-Wnt1* and *MMTV-Wnt10b* mouse models display precocious development of the lobular-alveoli so that the ductal termini display phenotypes similar to those usually observed during pregnancy in nonpregnant female and male mice [77,78]. These mice develop hyperplasias and adenocarcinomas at very high frequencies and with short latency. Transgenic mice with the *MMTV-LTR* promoter driving an activated form of β-catenin display a similar phenotype and support the notion that oncogenic WNT pathways operate via β-catenin [79]. One possible explanation for the aggressive tumour phenotypes observed is that the *MMTV-Wnt* tumours contain an expanded progenitor/stem cell population [21,80]. It has been suggested that WNT-induced progenitor amplification is likely to be key event in tumour initiation [81]. Novel therapeutic strategies could be developed by targeting pathways that modulate the progenitor populations in the mammary gland.

The *scaramanga (ska)* mutation is a useful model for elucidating the molecular mechanisms that govern specification of the mammary phenotype. The *ska* mutation impairs some of the earliest aspects of mammary gland development [82,83]. Bud 3 often fails to form or is hypoplastic, and ectopic mammary buds form adjacent to bud 4 at a high frequency. More subtle defects in mammary anlagen size, shape, and position are also observed so that the stereotypic position of the five pairs of mammary buds is rarely observed when mammary bud markers are used to visualize the embryonic buds. The mammary phenotypes observed in *ska* mutants are consistent with abnormal inductive events occurring prior to the morphological appearance of the mammary bud.

Positional cloning identified the gene affected in *scaramanga* (*ska)* mutants as *Neuregulin3* (*Nrg3*) [61]. Nrg3 is a poorly characterised member of an important signalling network and is expressed in some pre-invasive and invasive breast cancers [84,85]. *Nrg3* encodes a growth factor, which binds and activates the Erbb4 tyrosine kinase receptor [86]. Erbb4 regulates both cell proliferation and terminal differentiation in the mammary gland [87–89]. The preferred heterodimerisation partner for the Erbbs (including Erbb4) is Erbb2, which has profound links to breast cancer and which has been therapeutically targeted with positive clinical results [90]. Erbb4 also modulates cell migration in the developing nervous system. *Nrg3* is expressed in the rat forebrain along with many other Egf-related ligands, and neuroblast migration and placement within the rat forebrain is mediated by Erbb4 [91]. Erbb4 signalling controls Nrg1β1-induced migration in neural progenitor cells and also mediates the organization and proliferation of cells in the subventricular zone, the neurogenic region of the adult forebrain [92,93]. Another ligand for Erbb4, Nrg1, can induce migration of breast cancer and melanoma and cells in vitro [94,95]. It is plausible, therefore, that control of the migration of mammary epithelial precursors is modulated by Nrg-Erbb signalling.

Localised *Nrg3* expression in the presumptive mammary region prior to morphological appearance of buds and the expression of bud epithelial markers suggest an inductive role. Mammary anlagen appear at sites where *Fgf10, Tbx3,* and *Wnt* expression and *Nrg3*, *Erbb4* co-localize (i.e., along mammary line) in the lateral plate and overlying mesoderm (Figure 3). This coincident expression of *Tbx3, Fgf10, Nrg3,* and *Wnts* in the embryonic mesenchyme occurs just prior to the determination of embryonic ectoderm to mammary epithelial rather than remaining a simple epithelial fate. Ectopic mammary placodes can be induced in explant cultures by placing rNrg3-Egf–soaked beads adjacent to the dense mesenchyme along the mammary line that is marked by the expression of *Fgf10, Tbx3*, and *Wnts*. These results indicate that Nrg3 is a specification signal for mammary glands [61].

**Figure 4:**
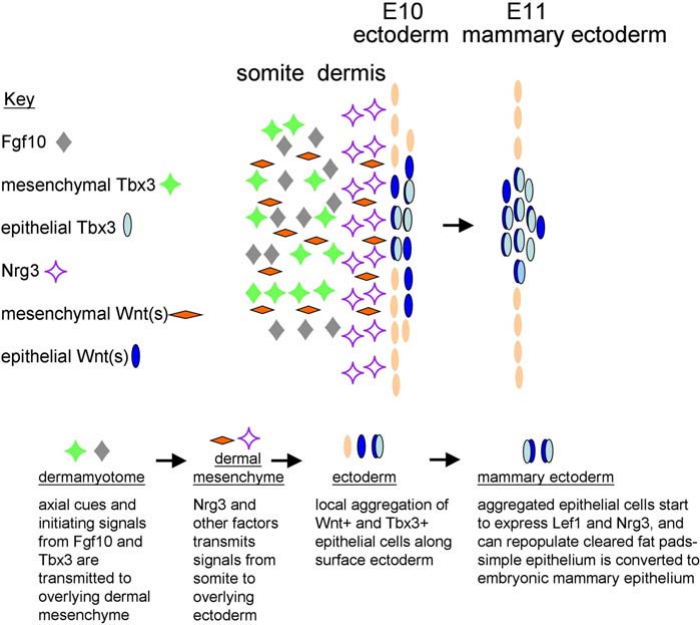
Model for Mammary Anlage Formation and Cell Fate Determination This model is based on functional studies as well as the expression patterns of *Fgf10, Nrg3, Tbx3,* and Wnts in the presumptive mammary region between E10 and E11 when initiating processes are likely to occur. Unknown axial cues are generated so that mammary anlagen arise at specific locations along the anterior–posterior, dorsal–ventral axis. Other factors are involved, and genetic requirements are likely to vary for each of the five pairs of anlagen. For example, *Fgf10* is not required for the initial formation of anlagen 4, so this simplified model applies to anlagen 1, 2, 3, and 5.

It appears that the inductive mammary line mesenchyme (which is the tissue from the presumptive mammary region that expresses *Tbx3, Fgf10,* and *Nrg3*) instructs mammary gland development when combined with other epithelia (which express *Fgfr2B* and *Erbb* receptors). How signals from Fgf10 and Tbx3 (and possibly mesenchymal Wnts) are transmitted from the lateral plate mesoderm to the precursor epithelial population is unknown, but Nrg3 is an attractive candidate to mediate this signal (Figure 4). At stages prior to the morphological appearance of the anlage, *Nrg3* is localised to the mesenchyme adjacent to the future site of the anlage (35–47 somite stage). At the stage that placode 3 is initially apparent by scanning electron microscopy (47 somite stage), *Nrg3* is first expressed at the basal edge of the anlage epithelia, and later all cells of the mammary epithelial bud proper express Nrg3. *Erbb4* and Erbb4 are expressed in a similar pattern. Erbb2 expression is also expressed in the early mammary anlage epithelia and mesenchyme before becoming restricted to the bud. Fgf10-soaked beads implanted into explanted mouse embryos had no effect on *Lef1* expression or epidermal morphology [15]. It is therefore likely that other factors are needed to transmit the signals generated by Fgf10. When Fgf8-soaked beads were implanted into explanted mouse embryos, increased expression of both *Tbx3* and *Lef1* were observed in the surrounding mesenchyme, but there were no morphological changes in the epithelium [53]. At the time the mammary anlagen are initially visible, *Tbx3* expression shifts from the mammary line mesenchyme to the epithelial component [53]. An increase was observed in both epithelial and mesenchymal Wnt signalling in Wnt reporter mice when rNrg3-soaked beads were implanted into explanted mouse embryos after 24 hours of culture [61]. In addition, epithelial aggregates are often found adjacent to the Nrg3-soaked beads, suggesting that ectopic Nrg3 expression can effect initiation of mammary anlage formation. These functional studies and the localization of *Nrg3* expression between the sites of *Fgf10* and *Tbx3* expression in the lateral plate mesoderm and the overlying Wnt-expressing epithelial cells support a model whereby Nrg3 transmits signals downstream of Tbx3 and Fgf10 to the overlying epithelia to effect their local aggregation [61] (Figure 4). Although useful as a hypothesis, this model is obviously simplistic, as other factors are clearly involved. Tbx3 and Fgf10 may affect Wnt signalling independently of Nrg3. The genetic hierarchies and precise relationships between Fgf10, Nrg3, Tbx3, and Wnts have yet to be fully elucidated, as is the role of each in the epithelial and mesenchymal compartments.

The differential effects of *Fgf10, Lef1,* and *Tbx3* deficiencies on both mammary anlage and ductal development of specific anlagen has been demonstrated [15,57,60]. How these and other genes contribute to the formation of specific anlagen along the body axis is not fully understood, but it is increasingly apparent that different genes regulate the initial formation of distinct anlagen [16]. The site between anlagen 3 and 4 appears to be very sensitive to growth factor levels, as demonstrated by studies of *Nrg3* and *Eda-A1* mouse models [96]. The temporal sequence of the initial expression of different genes in each mammary epithelia anlage is not identical, also suggesting that each may contribute to the initial formation of the anlagen along the body axis in different ways [15,53,57]. It is also not known how axial cues are transmitted to the future sites of the mammary anlagen. The expression of the major regulators of the very early stages of mammary morphogenesis is dynamic, and in the case of *Tbx3* and *Nrg3,* switch from the mesenchyme to the epidermis at the time the anlage is initially apparent. The change in expression may reflect a shift to distinct functions of these genes at later stages of mammary morphogenesis.

## Eda and Edar

Mutations in ectodysplasin pathway components result in ectodermal dysplasias in both humans and mice [97]. In addition to skin, hair, teeth, and sweat gland abnormalities, Hypohidrotic ectodermal dysplasia is sometimes associated with absent nipples [98]. *Tabby* and *downless* are mouse models with mutations in *Eda* and *Edar,* respectively. The initial formation of mammary anlagen appears to proceed normally in these models, although neither has yet been thoroughly analysed (Marja Mikkola, personal communication). The *K14-Eda-A1* mouse model displays ectopic mammary epithelial bud development and is likely to provide a molecular link between the results observed in the various mouse models described above, which display abnormal mammary induction [96,99]. In *K14-Eda-A1* mice, the five endogenous pairs of mammary buds develop in their stereotypic positions. Extra mammary buds and nipples also develop along the “mammary line” and mainly between the sites of buds 3 and 4. *Eda* is a target of Wnt signaling and Lef1 binds to a site within the *Eda* promoter, increasing the transcription of the gene [100]. *Eda-A1,* a splice variant whose product binds Edar, is thought to promote placodal fate as extra teeth and enlarged hair follicles are also observed in this mouse model. Signalling through Eda-A1 and Edar is mediated by NF-κB, which is frequently aberrantly activated in breast cancers [101]**.**

## Other Genetic Pathways

Many other genetic pathways contribute to early mammary anlage development. The epithelium fails to stratify and no mammary buds or other epidermal appendages form in *p63*-null mice [102,103]. p63 regulates several signalling pathways required for epidermal development. β*-catenin* and *Fgr2b* are downregulated in *p63*-null epithelium, which could account for impeded mammary morphogenesis [104]. A slight epithelial thickening occurs in mice in which both *Msx1* and *Msx2* are inactivated, but no distinct mammary anlage forms. Inactivation of either *Msx1* or *Msx2* alone does not affect anlage formation [105]. Both *Msx1* and *Msx2* can upregulate the expression of *CyclinD1* [106]. Notch signaling acts on mammary progenitor cells and promotes self-renewal and lineage-specific differentiation of myoepithelial fate [107]. Hedgehogs have also been implicated in self-renewal and maintenance of the mammary progenitor population [108]. Investigations of the roles of *Ihh* and *Shh* in mammary anlage development suggest that they are not required or that genetic redundancy may obscure the effects of loss of either [20,109]. *Gli3^xt^* mice, a null allele of *Gli3* (a transcription factor that forms a component of the Hh pathway), fail to form anlagen 3 and 5 [110]. Hh signaling appears to be blocked in *Lef1*-null mice as the expression of *Ptch1* (which is both an Hh receptor and a transcriptional target of Hh) is reduced in the mammary mesenchyme of *Lef1*-null mice [60]. A recent study of expression of SHH, PTCH1, and GLI1 in 52 breast cancers found that these Hh pathway components are frequently activated, when compared with adjacent normal tissue [111].

Nodal signaling regulates many developmental processes including differentiation, formation of the germ layers, and specification of the anteroposterior and left–right axes and of the embryonic midline [112]. Nodal acts through the TGF-β/ activin pathway by binding to Acvr2b, a type II activin receptor. Cripto acts as an essential cofactor for the Nodal pathway. *Cripto*-null mice lack a primitive streak and fail to form mesoderm [113], a phenotype shared by mice lacking *Acvr1b, Acvr2a, Acvr2b,* or *Nodal*. *CRIPTO* is overexpressed in breast, ovarian, gastric, lung, and pancreatic carcinomas [114]. Both *Cripto* and *Nodal* are expressed in undifferentiated human and mouse ES cells and are thought to promote maintenance of pluripotency [115,116]. Cripto and Nodal are both candidate mammary morphogens, although early embryonic lethal phenotypes have prohibited the examination of mammary anlage phenotypes [117].

It appears that a developmental window exists when the mesenchyme expresses the factors that are necessary to confer mammary epithelial fate on a select group of undifferentiated epithelia. It is unclear precisely how signalling pathways converge to elicit the mammary epithelia developmental program. Elucidation of the genetic hierarchy and mode of interaction of the various signalling molecules in the context of mammary specification will allow the integration of pathways known to be deregulated in human breast cancers [4,12,33,38,54,56,77,111,118–123].

## Conclusion

A better understanding of genetic pathways involved in early mammary gland morphogenesis is likely to have profound implications for breast cancer. Further delineation of the signals that initiate mammary differentiation should pave the way for the development of new therapeutic and preventative strategies. As more mammary stem cell markers are discovered and genetic models for early mammary gland morphogenesis are analysed, the question of how an undifferentiated epidermal cell can be selected to become the building block for one of the most fascinating organs will become closer to being answered.

## Supporting Information

### Accession Numbers

The National Center for Biotechnology Information EntrezGene (http://www.ncbi.nlm.nih.gov/entrez/query.fcgi?db=gene) accession numbers for the genes and proteins discussed in this paper are *Acvr1b* (11479), *Acvr2a* (11480), *Acvr2b* (11481*)*, *APC* (324), *AXIN2* (8313), β-catenin (1499), *CRIPTO* (6997), *Cripto* (12627), CyclinD1 (595), *CyclinD1* (12443), *dHand* (15111), *Dkk1* (13380), *Eda* (13607), Edar (13608), Erbb2 (13866), *Erbb4 (*13869), *Fgf3* (14174), *Fgf4* (14175), *Fgf8* (14179), *FGF10* (2255), *Fgf10* (14165), *FGFR1* (2260), Fgfr1 (14182), Fgfr2b (14183), *FGFR2* (2263), *FGFR4* (2264*)*, FZD1 (8321), FZD2 (2535), GLI1 (2735), *Gli3* (14634), *Ihh* (16147), *LEF1* (51176), *Lef1* (16842), Mdm2 (17246), *Mmp3* (17392), *Msx1* (17701), *Msx2* (17702), NF-κB (4790), Nodal (18119), NRG1 (3084), Nrg1(112400), *Nrg3*(18183), p14(ARF) (1029), p19Arf (12578), p53 (22059), p63 (22061), *Ptch1* (19206), PTCH1 (5727), *PthrP* (19227), *SFRP1* (6422), SHH (6469), *Shh* (20423), *TBX3* (6926), *Tbx3* (21386), *WIF1* (11197), *WNT1* (7471), *Wnt1* (22408), *WNT3A* (89780), *Wnt3a (*22416), *WNT4,* (54361), *WNT6* (7475), *Wnt6* (22420), *WNT8B* (7479), *WNT9A* (7483), *WNT10B* (7480), and *Wnt10b* (22410).
